# The Redesign and Validation of Multimodal Motion-Assisted Memory Desensitization and Reconsolidation Hardware and Software: Mixed Methods, Modified Delphi–Based Validation Study

**DOI:** 10.2196/33682

**Published:** 2022-07-12

**Authors:** Chelsea Jones, Lorraine Smith-MacDonald, Matthew R G Brown, Jacob VanDehy, Rasmus Grunnet-Jepsen, Vrajeshri P Ordek, Sarah Kruger, Anne Ayres Gerhart, Nancy van Veelen, Mirjam J Nijdam, Lisa Burback, Bo Cao, Michael J Roy, Pinata Sessoms, Eric Vermetten, Suzette Brémault-Phillips

**Affiliations:** 1 Leiden University Medical Center Leiden University Leiden Netherlands; 2 Heroes in Mind, Advocacy and Research Consortium Faculty of Rehabilitation Medicine University of Alberta Edmonton, AB Canada; 3 Alberta Health Services Edmonton, AB Canada; 4 Department of Warfighter Performance Naval Health Research Center San Diego, CA United States; 5 Leidos Inc San Diego, CA United States; 6 National Intrepid Center of Excellence Walter Reed National Military Medical Center Bethesda, MD United States; 7 ARQ National Psychotrauma Center Diemen Netherlands; 8 Department of Psychiatry Amsterdam University Medical Centers Amsterdam Netherlands; 9 Department of Psychiatry Faculty of Medicine and Dentistry University of Alberta Edmonton, AB Canada; 10 Department of Medicine and Center for Neuroscience and Regenerative Medicine Uniformed Services University Bethesda, MD United States; 11 Department of Occupational Therapy Faculty of Rehabilitation Medicine University of Alberta Edmonton, AB Canada

**Keywords:** multimodal motion-assisted memory desensitization and reconsolidation, multimodal motion-assisted memory desensitization and reconsolidation, 3MDR, participants, therapists, patients, military, virtual reality, mobile phone

## Abstract

**Background:**

In recent years, the delivery of evidence-based therapies targeting posttraumatic stress disorder (PTSD) has been the focus of the Departments of Defense in countries such as Canada, the Netherlands, and the United States. More than 66% of military members continue to experience symptoms of PTSD that significantly impact their daily functioning and quality of life after completing evidence-based treatments. Innovative, engaging, and effective treatments for PTSD are needed. Multimodal motion-assisted memory desensitization and reconsolidation (3MDR) is an exposure-based, virtual reality–supported therapy used to treat military members and veterans with treatment-resistant PTSD. Given the demonstrated efficacy of 3MDR in recently published randomized control trials, there is both an interest in and a need to adapt the intervention to other populations affected by trauma and to improve accessibility to the treatment.

**Objective:**

We aimed to further innovate, develop, and validate new and existing hardware and software components of 3MDR to enhance its mobility, accessibility, feasibility, and applicability to other populations affected by trauma, including public safety personnel (PSP), via international collaboration.

**Methods:**

This study used a modified Delphi expert consultation method and mixed methods quasi-experimental validation with the purpose of software validation among PSP (first responders, health care providers) participants (N=35). A team of international experts from the Netherlands, the United States, and Canada met on the web on a weekly basis since September 2020 to discuss the adoption of 3MDR in real-world contexts, hardware and software development, and software validation. The evolution of 3MDR hardware and software was undertaken followed by a mixed methods software validation study with triangulation of results to inform the further development of 3MDR.

**Results:**

This study resulted in the identification, description, and evolution of hardware and software components and the development of new 3MDR software. Within the software validation, PSP participants widely acknowledged that the newly developed 3MDR software would be applicable and feasible for PSP affected by trauma within their professions. The key themes that emerged from the thematic analysis among the PSP included the desire for occupationally tailored environments, individually tailored immersion, and the applicability of 3MDR beyond military populations.

**Conclusions:**

Within the modified Delphi consultation and software validation study, support for 3MDR as an intervention was communicated. PSP participants perceived that 3MDR was relevant for populations affected by trauma beyond military members and veterans. The resulting hardware and software evolution addressed the recommendations and themes that arose from PSP participants. 3MDR is a novel, structured, exposure-based, virtual reality–supported therapy that is currently used to treat military members and veterans with PTSD. Going forward, it is necessary to innovate and adapt 3MDR, as well as other trauma interventions, to increase effectiveness, accessibility, cost-effectiveness, and efficacy among other populations affected by trauma.

## Introduction

### Background

Military service commonly involves engagement in high-risk activities, whether during physical training, daily trade-related tasks, overseas deployment, or in response to natural disasters. Such activities place military members, individually and collectively, at a heightened risk of physical and psychosocial injury. They are also more likely to have increased exposure to highly traumatic and stressful events [1] and exhibit higher rates of injuries and illnesses, such as posttraumatic stress disorder (PTSD), depression, anxiety, sleep disorders, and mild traumatic brain injury compared with civilians [2,3]. In Canada, approximately 11% of military members and 16% of veterans experience PTSD [4]. In the United States, the prevalence of combat-related PTSD ranges from 1.09% to 38.84% [5]. The rate of probable PTSD among UK military personnel has been reported to be 6.2%, and among veterans who had been deployed in combat roles, it was 17.1% [6,7]. In Australia, approximately 8.3% of Defense Force members will have experienced PTSD [8]. Among currently serving and retired military personnel in New Zealand, 30% had symptoms of posttraumatic stress, and 10% had clinically relevant posttraumatic stress [9].

Hypervigilance, impaired cognition, comorbid mood and anxiety disorders, and significant functional and relational sequelae are associated with PTSD [1]. Military members and veterans are also more prone to developing moral injury than civilians, which is the persistent distress that can evolve from exposure to potentially morally injurious events, including perpetrating, witnessing, or failing to prevent an act that transgresses core beliefs [10-13]. Up to 67% of military members are exposed to potentially morally injurious events during deployment, the sequelae of which have not yet been fully explored, although it has been suggested that moral injuries might contribute to treatment-resistant PTSD (TR-PTSD) [14,15].

### Trauma-Focused Therapies and TR-PTSD

The delivery of evidence-based therapies specifically targeting posttraumatic stress throughout the military health care system has been the focus of the Departments of Defense in numerous countries, including Canada, the Netherlands, and the United States. Greater than 66% of military members who complete first-line treatments for PTSD continue to experience symptoms that significantly affect their daily functioning and quality of life [16]. Even in those responsive to treatment, PTSD symptoms often persist at or above the diagnostic thresholds for PTSD, with approximately 60% of patients retaining the diagnosis [17,18]. The effectiveness of these first-line psychotherapies is hypothesized to be limited because of cognitive avoidance and premature treatment dropout [19,20]. The reasons for higher dropout rates among this population vary but may include geographical proximity to services, perceived stigma from self and others, challenges managing operational demands of a military career with therapy attendance, barriers to establishing patient-therapist rapport, and the aforementioned issues with treatment effectiveness [19,20].

The classification of TR-PTSD has been adopted for military members and veterans who do not experience a clinically significant reduction in symptoms following the receipt of at least two evidence-based treatments [21,22]. As knowledge of TR-PTSD is limited, general recommendations for TR-PTSD have been suggested. However, specific protocols or evidence-based TR-PTSD therapies are lacking, complicating clinical attempts to address or manage this condition. Consequently, more innovative and effective treatments are needed to assist military members, veterans, and other populations affected by trauma in rehabilitation and recovery from PTSD and occupational stress injury.

### Multimodal Motion-Assisted Memory Desensitization and Reconsolidation

Multimodal motion-assisted memory desensitization and reconsolidation (3MDR) is a personalized, exposure-based, virtual reality (VR)–supported intervention developed in the Netherlands and is being used with military members and veterans with PTSD in the Netherlands, the United Kingdom, the United States, Israel, and Canada [23]. 3MDR enhances visual and auditory immersion by incorporating patient-selected images and music into the therapy and helps patients access traumatic memories as they walk toward images that remind them of those experiences [24]. The treatment shows promise for breaking through persistent avoidance and optimizing engagement in military members and veterans with TR-PTSD [25,26]. Two recently published randomized controlled trials conducted with veterans with TR-PTSD have contributed to the evidence base regarding the effectiveness of 3MDR [27,28]. Further studies are also underway.

For 3MDR, treadmill walking on a flat surface was chosen as the method of moving forward (literally and metaphorically) versus using other devices, such as a stair climber, which involves other movement patterns and requires more engagement of energy systems and cardiorespiratory abilities. As walking is a basic form of human movement that engages multiple processes and areas of the brain, it has been hypothesized that the act of moving forward through ambulation is integral to 3MDR and may increase divergent thinking abilities, thereby allowing previously held negative cognitions related to trauma to be challenged. In addition, a harness can be easily attached and added to a treadmill setup, making it a safer option than using other movement devices or virtual boundaries (ie, VR or augmented reality [AR] headset systems such as Oculus, Google Glass, or Microsoft’s HoloLens).

**Figure 1:**
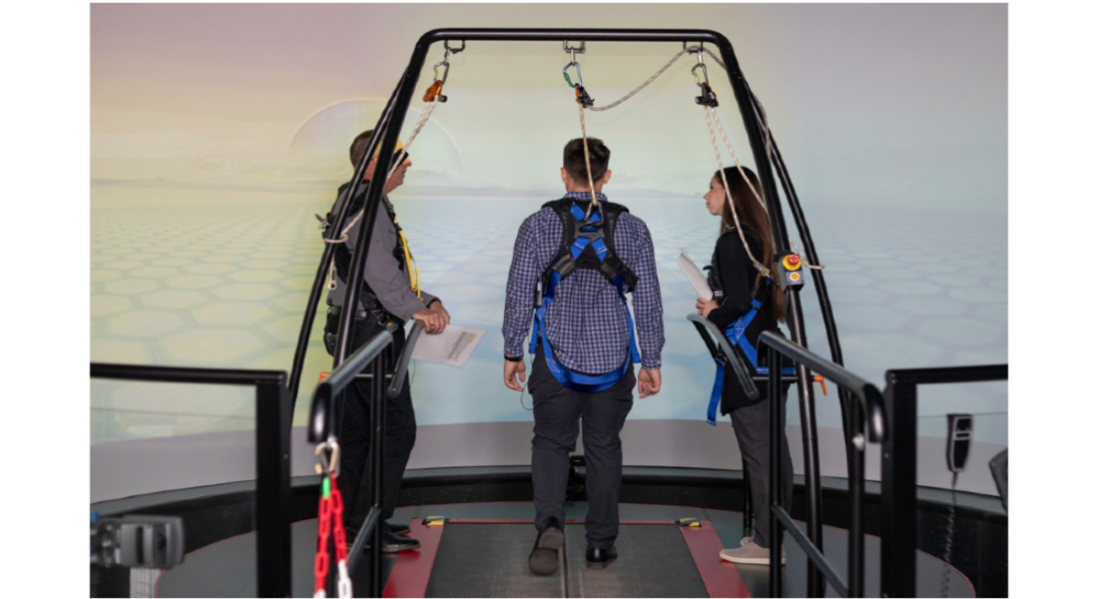
Multimodal motion-assisted memory desensitization and reconsolidation therapists and a participant in the Computer Assisted Rehabilitation Environment system.

The 3MDR application was originally designed to be used on a large-scale, immersive VR–based system called the Motek Computer Assisted Rehabilitation Environment (CAREN; Figure 1) and the Gait Realtime Analysis Interactive Lab (GRAIL; Motek Medical BV) [29]. These were designed as dedicated solutions for gait analysis and training under challenging conditions to improve gait patterns. CAREN is a room-sized dynamic system that includes a motion platform that can translate and rotate in all directions. The platform contains an embedded, centrally located treadmill that moves in synchronization with the virtual environments projected onto a large curved screen. The package contains real-time feedback and user-friendly assessments and applications. The GRAIL originally used an instrumented dual-belt treadmill with optional pitch and sway, a motion capture system, video cameras, and synchronized VR environments (VREs). The treadmill’s ability to measure forces and the independent control of left and right treadmill belt speeds has allowed a split-belt walking protocol and advanced gait research applications that can mimic tripping or slipping. The 3MDR application has progressively evolved to include other hardware options (ie, CAREN Lite) since the initial proof of concept was piloted in 2011 [23]. These versions of 3MDR hardware allowed for preliminary research to assure participant safety with clear, tangible boundaries as opposed to virtual ones, as experienced within the VR or AR headset systems. This was especially important at the time of the intervention’s developmental process when there were still many unknowns about the participant’s experience of 3MDR and the responses it may elicit.

The 3MDR intervention comprises 10 sessions, including selecting images and music, trauma processing and reconsolidation, and six 90-minute therapy sessions in the VRE, including a 30-minute debriefing. The 3MDR sessions include a *preplatform* *session* (session 1), during which the patient selects and orders images and music. Symbolic representations in the form of images (ie, photographs and sketches) related to their traumatic experiences are selected and ordered from least to most distressing. Music that reminds the patient of the past time of trauma and facilitates the emotional memory network is also identified, which supports a return to the present (ie, a second contemporary piece that is soothing, compassionate, and joyful). Sessions 2 to 7 are *platform sessions* that involve 3 phases. In the *preplatform phase* of the session, the therapist and patient confirm the order of images and music for the session. During the *platform phase*, the patient dons a safety harness and is accompanied by a 3MDR therapist while walking continuously on a treadmill at a self-selected pace. The patient first warms up by walking on the treadmill while listening to self-selected music, which connects them to their traumatic experiences, and then, during each of seven 3 to 5 minutes cycles, walks down a virtual *hallway* toward a self-selected trauma-related image. The patient describes the image, physical sensations, and feelings to the therapist. The therapist then assists the patient with generating descriptive words and phrases that are then projected in front of the image on the screen. The patient is then instructed to read the words and phrases out loud. For a duration of 30 seconds, the patient then reads aloud numbers as they appear on a ball oscillating horizontally in the foreground of the image and words. The patient cools down after the seventh cycle by walking while listening to self-selected music, which facilitates a reconnection to the present. Each session concludes with a *postplatform phase*, which includes discussion, reconsolidation, a mental wellness check, and a self-care plan. *Postplatform session*s 8 to 10 focus on reconsolidation and contribute to meaning making of the acquired gains and emotional release. More in-depth descriptions of 3MDR have been published elsewhere [25,30].

### Challenges With the Current Iteration of 3MDR

Recently, published randomized controlled trials have demonstrated the efficacy of 3MDR therapy among military and veteran populations who experience combat-related TR-PTSD and indicate that 3MDR is ready for trials with various populations affected by trauma in real-world contexts [27,28]. However, access to the CAREN VREs used to deliver 3MDR in earlier studies is limited because of the cost and infrastructure required by these large systems and the reality that only limited international partners in the Netherlands, Tel Aviv, the United States, the United Kingdom, and Canada have access to equipment currently used to deliver the intervention. 3MDR systems that are mobile, customizable, accessible, cost-effective, and adaptable to other populations affected by trauma are needed to increase accessibility to treatment. There is a sense of urgency in the 3MDR international consortium to develop further systems, particularly in light of their need within military and veteran populations, as well as among other populations affected by trauma. The COVID-19 pandemic has acted as a reminder that emergency medical professionals and first responders, among others, also require access to innovative and effective evidence-based treatments.

Despite the urgent need for accessible interventions for PTSD, a barrier to the expanded access to 3MDR in community-based health service contexts is the use of equipment and technologies approved by the United States Food and Drug Agency and Health Canada. Although these approvals are imperative to protect the health and safety of patrons of health care systems, the approval process requires the determination of appropriate hardware (eg, treadmills, projection systems, and AR equipment) and the development of software that is adaptable for various populations. Provision of research and evidence demonstrating equipment validity, safety, efficacy, and effectiveness specific to the target demographic is the first step toward improving access to novel interventions with technological applications. Now that research has demonstrated that 3MDR holds promise, new research may involve the exploration of adaptations including the delivery of 3MDR via more mobile, affordable, and immersive mechanisms, such as AR headsets, and to additional populations affected by trauma.

### Purpose

The purpose of this mixed methods study was to further innovate, develop, and validate new and existing hardware and software components of 3MDR to enhance the mobility, accessibility, feasibility, and applicability of 3MDR for other populations affected by trauma, including public safety personnel (PSP), via international collaboration.

## Methods

### Project Design and Scope

This mixed methods study used a (1) modified Delphi expert consultation method and, (2) a mixed methods quasi-experimental software validation study [31].

#### Modified Delphi Expert Consultation

##### Overview

The team of international experts from the Netherlands, United States, United Kingdom, and Canada comprised health care professionals (psychiatrists, psychologists, and occupational therapists), biomedical and electrical engineers, computing science experts, and graphic designers with prior experience in working with populations affected by trauma and the 3MDR system. The team met virtually on the web on a weekly since September 2020 to discuss the current and future development of 3MDR. Discussions revolved around hardware design, software design and validation, adoption of 3MDR in real-world contexts, the use of 3MDR with nonmilitary and veteran populations affected by trauma, and 3MDR therapist training. This involved consideration of the various systems that have been used for 3MDR, as well as salient features, emerging technologies, and potential future states. This manuscript focuses solely on the hardware design and software development and validation, which are described in the following section, whereas the implementation of 3MDR in community-based service contexts and training of 3MDR therapists will be included in future publications and an upcoming 3MDR training manual.

##### Considerations for Hardware Design

The 3MDR system requires the components described in Textbox 1. The 3MDR system must have the characteristics described in Textbox 2.

**Multimodal motion-assisted memory desensitization and reconsolidation system components.**

**Treadmill**

- The treadmill must be a medical-grade, clinical treadmill designed for heavy use over long periods and with patients of varying body types and compositions. The belt moves to the rear, requiring the user to walk at a speed matching the belt. The rate at which the belt moves is equal to the rate of walking. The speed of walking needs to be controlled and measured.

**Safety harness**

- A safety harness is required to prevent potential injuries due to falls. An option is to support it using a connector hanging from the ceiling. Other support options include a frame setup around the treadmill or a gantry built into the treadmill.

**Visual display**

- The visual display must be a sufficiently large screen that allows the display of images in a way that maximizes immersion. The display can be a series of mounted computer monitor screens or projection screens combined with short-throw projectors. Typically, to allow immersion, the screens must surround the person to enable the feeling of walking in the virtual reality environment. An augmented reality (AR) system provides another alternative to monitor- or projector-based display systems. Unlike a virtual reality headset, an AR headset (eg, Microsoft HoloLens 2 and Magic Leap) is neither fully opaque nor fully immersive [32]. The AR headset superimposes a digital display over the participant’s regular visual field, providing a blend of virtual and real visual stimuli. AR allows patients to walk confidently as they can still see real-world anchors. The patient and therapist can also see each other, which is important for rapport during therapy and to allow the therapist to read the patient’s facial cues.

**Computer**

- The computer must have graphics processing capability to drive the display system without noticeable frame dropping, which would create distracting “jerkiness” in the display.

**Treadmill or computer interface**

- There must be a means of synchronization between the treadmill’s motion and the motion of the patient in the virtual environment. Options include direct control of the treadmill by a computer to match the movement in the virtual environment or sensing of the treadmill’s motion by a speed sensor, which is then used to control the movement in the virtual environment.

**Eye-tracking capability**

- The ability to capture eye movement and visual avoidance or focus using eye-tracking equipment was considered.

**Multimodal motion-assisted memory desensitization and reconsolidation (3MDR) system characteristics.**

**Access**

- It is desirable that the system accommodates being located in community-based contexts and standard clinical settings, possibly with space limitations and noise considerations.

**Physical footprint**

- The system must have a relatively small physical footprint such that it can be situated in standard rooms in clinical settings without extensive renovations.

**Portability**

- Portability may be desirable for certain clinical applications (eg, mobile 3MDR operations).

**Therapist positioning**

- The final system must allow the therapist to stand beside the patient. Discussions with patients in previous studies have indicated that this positioning is important, with patients expressing distaste for the therapist being behind or in front of them.

**Operation of the system**

- The final system would best be managed by the therapist, without the need for a dedicated 3MDR operator, so as to enhance therapist control over the session and reduce personnel costs.

**Cost**

- Affordability is a concern as we work toward the deployment of 3MDR systems more widely in clinical settings outside of research environments. We are currently exploring the use of off-the-shelf components to reduce costs.

**Acceptability or ease of use**

- The system should be easy for therapists to set up, use, and adapt. It should also be able to record data and enable note-taking while therapists deliver the intervention. Visualization of changes over time is also essential to facilitate treatment planning.

##### Software Design

The original 3MDR software was developed by a team in the Netherlands using the D-Flow software [29] in combination with Lua scripting. Our team of clinical and technical experts identified salient features of the original 3MDR software, as well as aspects that would benefit from redesign or further development. The new 3MDR software development was completed in the Warfighter Performance Department, Naval Health Research Center, San Diego, California, United States. As all components of the 3MDR intervention were already included in the D-Flow version, these essential components were maintained for consistency, although optimized. These included a user interface (UI) and 2 distinct visual scenes. The UI contained application settings, image selection options, and a drop-down menu to record subjective units of distress scores, which were measured as part of the 3MDR therapy. Examples of application settings include the frequency of eye movement desensitization and reprocessing (EMDR) stimuli, which are incorporated within 3MDR therapy, and treadmill speed. The 2 visual scenes included the following:

- Warm-up or cool down scene: bright lighting and white ground with blue honeycomb overlay; open, flat, and endless
- Corridor scene: darker lighting and dark ground with red honeycomb overlay; starts with doors at a distance, which opens to the first of 2 enclosed hallways with another set of doors at a distance, which open to a second hallway at the end of which the image related to a traumatic event is visible

##### Considerations for Software Design

The new 3MDR software required the components described in Textbox 3.

**New multimodal motion-assisted memory desensitization and reconsolidation system components.**

**Ease of adaptation to new technologies**

- The new system needs to be built on a cross-platform game engine that allows easy adaptation to new technologies and that can be ported to different devices, displaying the same visuals on different types of screens or through head-mounted displays.

**User interface**

- The new user interface should be therapist centered and user-friendly so that both operators and therapists feel confident using the application. The primary therapist interface, which mirrors the participant view, should allow the application to be configured and launched. The interface should allow session information to be input (eg, identification number, protocol number, and therapist initials), image and data folders to be selected, and site-specific settings to be chosen. Once the application is launched, secondary application-specific interfaces should appear on the operator or therapist display to control different aspects of the virtual environment. Other critical considerations include enhancing the therapist’s ability to dynamically select and modify the order of the images and music; adjust the motion and speed of the oscillating ball for eye movement desensitization and reprocessing; capture and record the emotional, cognitive, and narrative associations; and record subjective units of distress scores as well as therapist notes.

**Music selection**

- Audio features must be built into the software to support the integration of patients’ self-selected music within the multimodal motion-assisted memory desensitization and reconsolidation protocol.

**Data capture and reports**

- The system must provide clinical output sheet summaries generated on a per-session basis, as these are vital for treatment planning, case review, and research purposes.

#### Mixed Methods Quasi-Experimental Software Validation Study

After the adaptation and redesign of the software and hardware, a validation study was implemented with the goal of ensuring the face validity of the new version of the 3MDR software for health care professionals and first responders. We used a mixed methods, concurrent, parallel approach following a data transformation model [31].

##### Participants

Potential participants were recruited through snowball sampling and advertising via email, social media (Facebook and Twitter), hardcopy posters in targeted work environments, and word of mouth. Potential participants were asked to positively enroll via emailing or texting the research team. Eligible participants had to be health care professionals or first responders, including police officers, firefighters, border patrol officers, nurses, physicians, physical therapists, occupational therapists, crisis management workers, corrections officers, emergency medical personnel, dispatchers, respiratory therapists, psychologists, psychiatrists, and other professionals or managers associated with these professionals within a health care setting. Participants had to be able to communicate in English; live in Canada; and have access to a digital device such as a computer, tablet, or smartphone where videoconferencing was possible.

##### Data Collection

Questionnaire data were collected and managed using the secure REDCap (Research Electronic Data Capture; Vanderbilt University) system [33,34]. The demographics questionnaire gathered information on participants’ gender, age, and profession. The validation questionnaire included Likert scale questions and open-ended questions regarding various aspects of the software displayed in the demo video (ie, overall appearance, beginning scene, transition through the clouds, the scene around the first door, ground grid, hallway, image size, ball and movement, picture fade, transition to second door and hallway, timing, visual field, and flow), the impact of 3MDR on medical condition, ease, clarity, understanding of interaction with the 3MDR system, and ease of use of the system. The validation questionnaire was developed via discussion and collaboration among 3MDR international consortium researchers in Canada, the Netherlands, and the United States.

Focus group interviews were used to collect qualitative data for the validation study. Zoom videoconferencing was used. Members of the research team received training on how to execute the focus group for fidelity and consistency. A semistandardized script (Multimedia Appendix 1) was developed to further improve the reliability. Eligible participants were scheduled for 1 of the 25 researcher-led videoconferencing focus groups in July 2021. Each focus group lasted approximately 20 minutes and had attendance ranging from 1 to 12 attendees. The researcher leading the focus group proceeded as follows: (1) provided an introduction to 3MDR via 2 videos developed by the 3MDR team in the United Kingdom, (2) provided a weblink to the web-based informed consent form through the REDCap system, (3) displayed a 5-minute demo video of the adapted 3MDR software that participants were evaluating, and (4) provided a weblink to the demographics and validation questionnaire presented in REDCap. After completing the forms, the participants left the videoconference.

##### Data Analysis

Descriptive statistics were computed for demographic characteristics and Likert scale questions regarding various aspects of the software demonstration. For a given question, the number and percentage of different answer choices selected across the participants (N=35) were computed.

The open-ended questions in the validation questionnaire were thematically analyzed by the research team using the deductive and inductive methodology outlined by Braun and Clarke [35] in 2006. Thematic analysis involves examining the text in detail to identify recurring patterns (open coding) that are refined into themes through inductive and deductive analysis. Although deductive coding was guided by the preconceived research questions in the validation questionnaire, inductive coding was used to examine the participants’ views while limiting researchers’ preconceived biases and expectations. A total of 3 members of the research team read all open-ended responses and developed preliminary open codes and axial codes. To ensure trustworthiness, rigor, and reliability, a larger research team was involved in verifying the preliminary themes. Participant quotes illustrating each subtheme were selected based on their representativeness and the incidence of divergent opinions.

A concurrent parallel approach following a data transformation model was used for data triangulation [31]. Converging data allowed the research team to compare and contrast quantitative and qualitative data and support the subsequent study design, data collection, and analysis.

### Ethics Approval

This study was approved by the University of Alberta Research Ethics Board (Pro00084466) as part of a Canadian 3MDR study [30].

## Results

### Hardware Specification

Hardware design based on expert consultation produced designs for two 3MDR systems: one using an external display (3MDR compact system) and the other using an AR headset (3MDR AR system). Both systems are described in the following sections.

#### 3MDR Compact System

**Figure 2:**
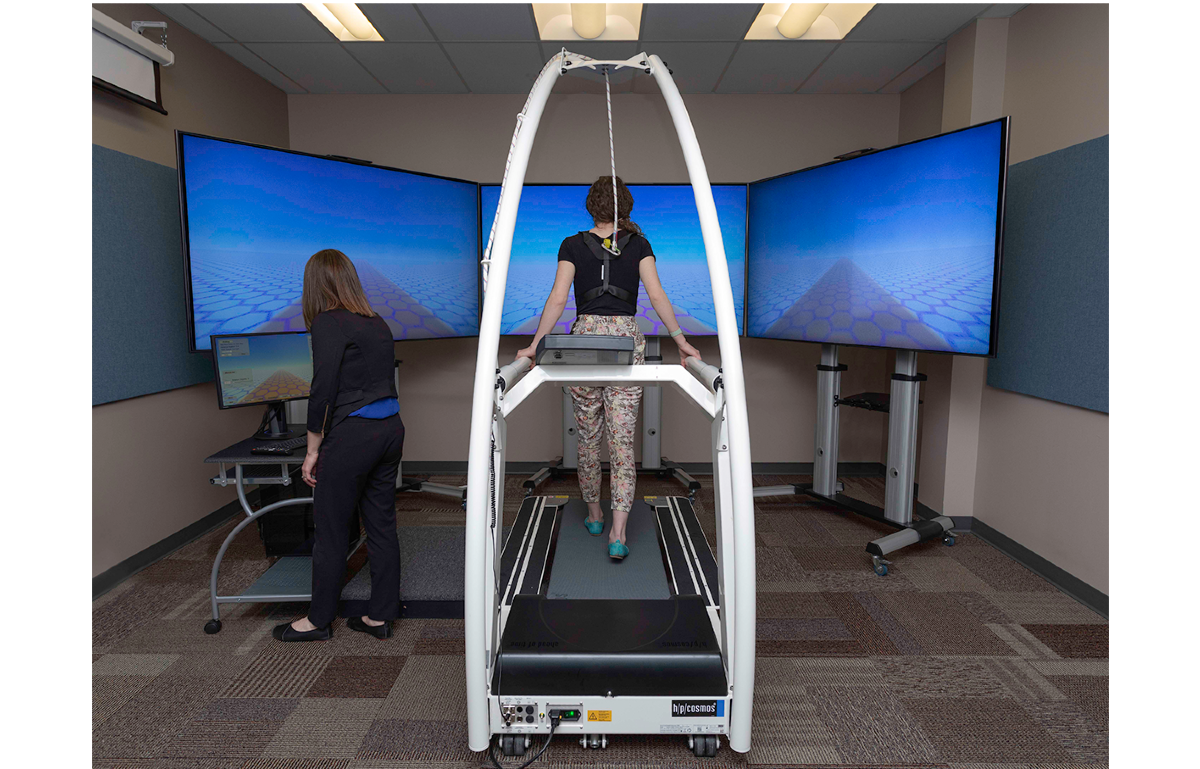
Compact system of the multimodal motion-assisted memory desensitization and reconsolidation.

**Figure 3:**
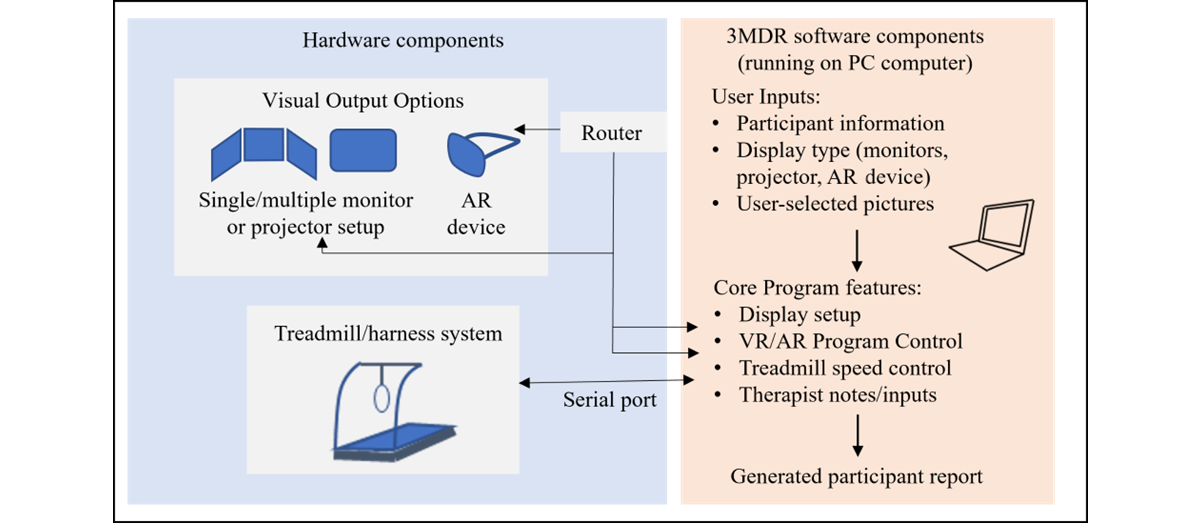
Schematic diagram of the hardware and software components of the compact system and future integration of an AR system. 3MDR: multimodal motion-assisted memory desensitization and reconsolidation; AR: augmented reality; VR: virtual reality.

The 3MDR compact system (Figures 2 and 3) was designed to have a smaller physical footprint than the large COSMED system used in earlier 3MDR studies. We identified the COSMED T150 clinical treadmill as a viable option around which to build the 3MDR compact system [36]. The COSMED T150 is a heavy, robust treadmill designed for clinical use in physical rehabilitation, with a maximum load rating of 250 kg (551 lbs) and variable speed options up to 18 km per hour (11.2 miles per hour). It has a built-in safety harness and gantry to support the harness. This makes installation easier and avoids the need for other solutions, such as a separate frame around the treadmill to support a harness or a harness support system installed on the ceiling of the room. The COSMED T150 has a serial port interface capability that allows a computer to control it programmatically, which enables synchronizing treadmill motion with movement in the virtual environment. The treadmill, with medical certification (C0123, Medical Device Directive risk class IIb) available for clinical applications, has a durable belt (including a reverse option) and an alternating current motor designed to not interfere with other medical equipment and can be interfaced with a PC, electrocardiogram, ergospirometer, blood pressure, or printer. A wide range of options and accessories are available for the heavy-duty–built treadmills in the series, including oversized treadmills for cycling or skiing applications and wheelchairs. The units also reportedly require low maintenance.

For the control computer, the research team in San Diego used computers built around the Nvidia Quadro P5000 graphics card [37], with 3 Canon WUX500_WUX450ST projector setups to project a blended image using Scalable Display Technologies’ Warp and Blend system [38,39]. The research team in Edmonton, Alberta, Canada, is currently looking into a similar approach using computers built around the Nvidia Quadro RTX 6000 graphics card and short-throw light-emitting diode projectors [40].

#### 3MDR AR System

**Figure 4:**
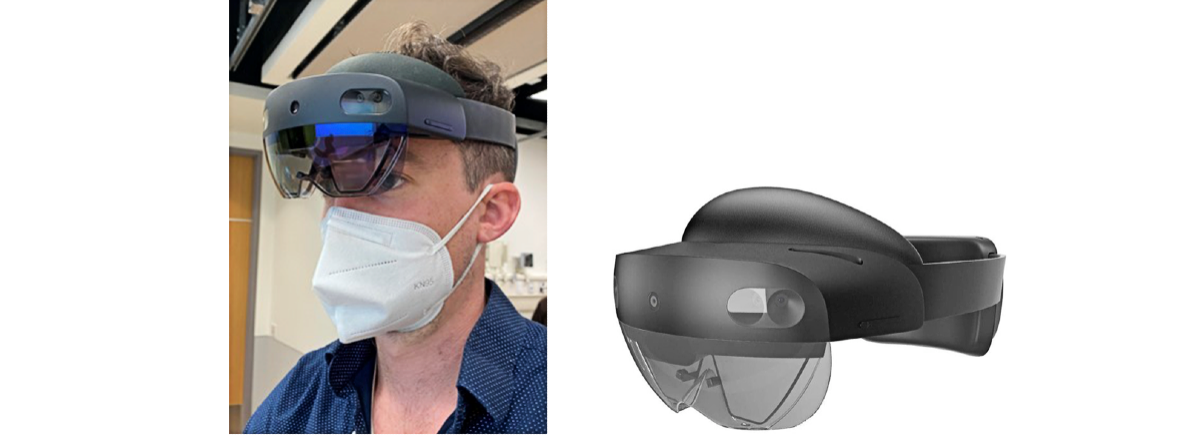
HoloLens 2 augmented reality system.

The team determined that the AR system would use a HoloLens 2 system (Figure 4) for the visual display in place of large computer monitor screens or projection systems [32]. The 3MDR AR system includes a laptop, router, treadmill, and HoloLens 2 headset. There are 3 reasons why HoloLens 2 was chosen over the other devices: safety, rapport, and eye-tracking capability.

Walking in HoloLens 2 versus other more immersive and isolating VR headsets posed less risk for our participants. Walking was required throughout the duration of each session, and the AR head-mounted display (HMD) was less of a safety risk as users could actively look down and see their feet on the treadmill. In addition, many VR HMDs are tethered devices that require cables connecting the HMD to a computer. These cables can be viewed as tripping hazards and restrict movement to a limited area, which would be prohibitive for 3MDR where walking is a key component. Although VR headsets might have provided greater immersion in the virtual environment, walking is an essential component of the 3MDR therapy.

In other 3MDR publications [41], it has been noted that the connection between the therapist and client is an important aspect of 3MDR. It is beneficial for the participant to be able to see the therapist alongside them for guidance and reassurance. The AR HMD does not restrict users from seeing their therapist during 3MDR. Rapport is key for this approach, and an AR HMD allows us to provide digital content in front of the user (and we can lock the content at that location). Therefore, when they turn to their side, they turn away from the 3MDR environment and can see their therapist unobstructed through the device, akin to looking through sunglasses. In a VR HMD, turning one’s head would only show users a different view of the virtual environment. On the basis of participant feedback, it was decided that an avatar (digital representation in the virtual world) of the therapist would not suffice as a stand-in, especially when AR allows users to see the real person and digital content.

### Software Redesign

#### Overview

The new software for 3MDR was developed by a research team in the Warfighter Performance Department, Naval Health Research Center, San Diego, United States. To allow for use across different hardware platforms, the Unity development platform was chosen to develop a new controlled version of 3MDR for both PC and HoloLens 2 systems (Figure 4) [42]. The new version of 3MDR was redesigned in Unity 3D, a cross-platform game engine that allows easy adaptation to new technologies. Unity 3D is an open-source C#-based game development platform or program commonly used for game and simulator development, which allows for deployment to multiple platforms. Unity 3D allows for enhanced graphics and physics, as well as programmatic flexibility so that applications can be modified and can evolve over time. The visual scenes were modified to achieve higher resolution and provide greater realism based on therapist feedback on the earlier D-Flow version of the 3MDR software. The Unity 3D platform is very extensible and handles deployments to a large number of software bundles, including PC stand-alone, Universal Windows Platform, and the Apple standard iOS [43]. Similarly, it allows flexibility in integrating heart rate monitors, eye trackers, and other wearable devices for future use. The design of this program emphasizes *modularity* to integrate and subtract devices for research and therapeutic purposes and *polymorphism* to deploy to a variety of current and future devices. The 3MDR application can be ported to different devices, displaying the same visuals on different types of screens or through HMDs.

#### Two Views: Therapist and Patient

**Figure 5:**
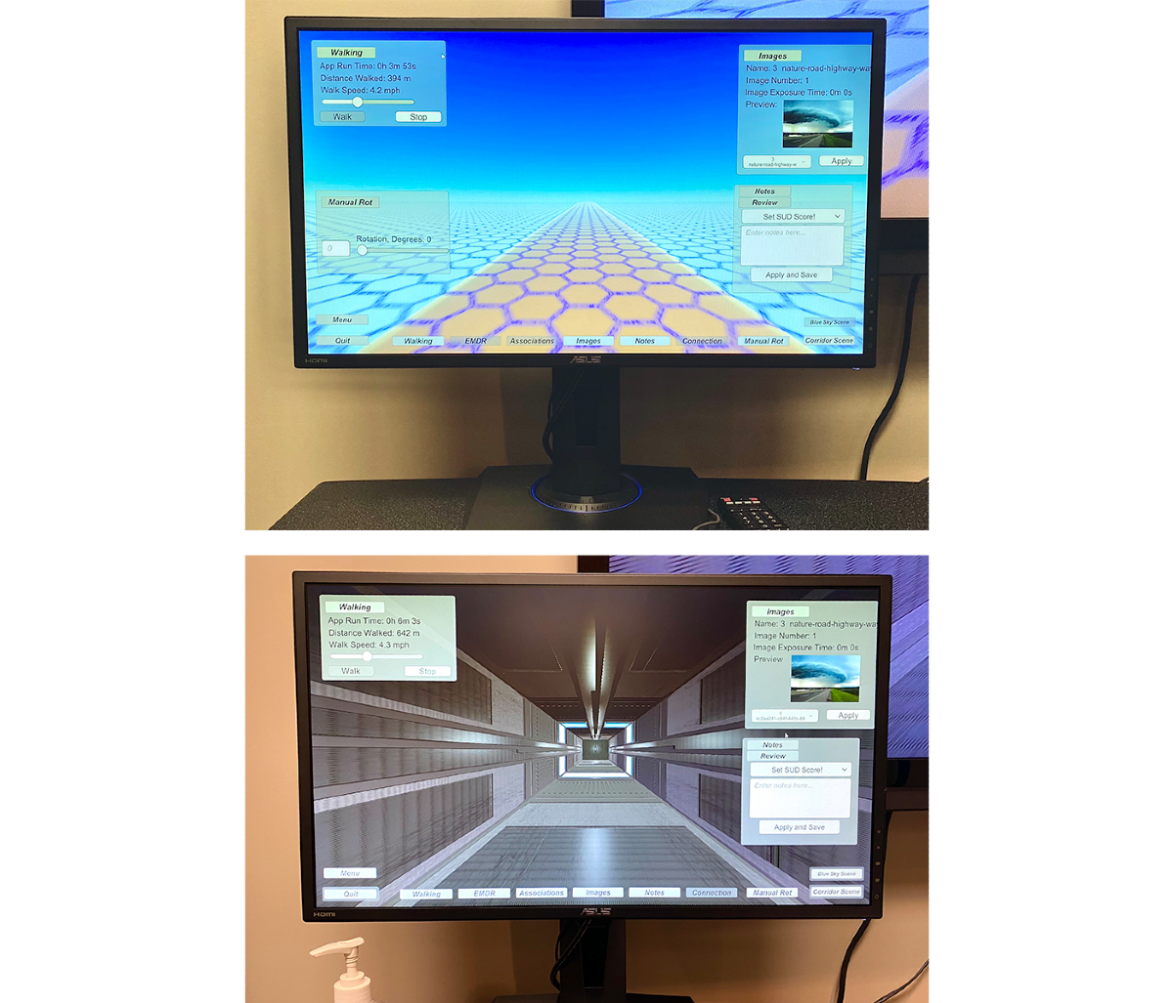
Therapist view on a PC.

This application has 3 scenes: introduction, corridor, and conclusion. Each has a different configuration for the patient and therapist. The introduction and conclusion are in the same environment, in which a blue sky celestial environment is used to transition the participant into and out of the therapeutic environment using self-selected music discussed with the therapist. The corridor scene contains a self-selected series of images previously selected during the discussion with the therapist. Visual flow from the simulation is synchronized directly to the treadmill motion to immerse the patient in therapy. The therapist’s view (Figure 5) overlays the control and data entry UI atop the mirrored visual flow. The following are all entered and automatically saved during the application process: walking speed; patient disposition notes and subjective units of distress scores; and emotional, cognitive, and narrative associations. Data are saved in a text file for postsession review. After establishing high involvement with the traumatic memories being worked through, a disengaging working memory task analogous to EMDR is displayed. An oscillating red ball is presented for a duration of 30 seconds, moving from left to right in the foreground of the screen. The patient reads aloud the superimposed number on the red ball. The purpose is to enable the patient to learn to disengage from traumatic memories by tapping into another brain circuit in a similar way to EMDR. After 30 seconds, the picture fades, and the scene transitions to a new cycle. Between cycles, the patient can take time to decompress through battle breathing exercises, drinking water, and reorienting to the new upcoming cycle. Therapists can manipulate the presentation and timing of these tasks and visuals to adapt to the patient’s response to therapy.

This final interface design was created through active consultation with therapists practicing 3MDR using the previous Motek design. Input was sought through bimonthly demonstrations of both the VRE and UI. Modifications were made to minimize UI complexity; mirror the view of the participant to the control system; and create summaries of each session, which can be readily reviewed by therapists following sessions for clinical notation.

#### Display Options

The current application supports deployment to either three-screen or HoloLens 2 for patient display. The 3-screen setup supports either a Scalable Display Technologies warp and blend for projected screens or independent television screens. The HoloLens setup uses the Photon Networking system (Exit Games) to pass information between the therapist controller and the patient viewport [43]. The visual flow is updated based on the walking speed and dynamically compensates for the ping rate by the Photon engine. Data are saved locally on the therapist control computer for both the 3-screen and HoloLens 2 displays.

#### Adaptation to Specific Patient Groups

Although it was originally thought that specific changes and adaptations would need to be made for various populations affected by trauma, it was determined that self-selected images and music rather than changes to the foundational elements of the 3MDR environment were required for tailored immersion.

### Software Validation

The quantitative and qualitative study findings in the subsequent sections reflect participant feedback (N=35) regarding the new 3MDR software.

#### Demographics

Table 1 shows the demographics for participants (N=35) recruited in Edmonton in July 2021.

**Table 1:** Demographics of the participants (N=35).

Demographics			Values, n (%)
**Gender**			
	Woman	22 (63)	
	Man	12 (34)	
	Rather not say	1 (3)	
**Age (years)**			
	20-29	4 (11)	
	30-39	13 (37)	
	40-49	13 (37)	
	50-59	3 (9)	
	≥60	1 (3)	
	Unknown	1 (3)	
**Role or designation**			
	Police	5 (14)	
	Firefighter	2 (6)	
	Correctional worker	2 (6)	
	Registered nurse	4 (11)	
	Support worker	3 (9)	
	Respiratory therapist	4 (11)	
	Psychologist	4 (11)	
	Medical physician	1 (3)	
	Manager	1 (3)	
	Social worker	4 (11)	
	Clinical social worker	2 (6)	
	Exercise specialist	1 (3)	
	Care provider for older adults	1 (3)	
	Other	1 (3)	
**Supported COVID-19?^a^**			
	No	2 (6)	
	Yes	31 (89)	
	Unknown	2 (6)	

^a^Indicates the number of participants who provided support and services during the COVID-19 pandemic.

#### Quantitative Results

Table 2 shows histogram results for Likert scale questions asking participants about various aspects of the 3MDR video. Most participants liked most aspects.

**Table 2:** Distribution of responses to Likert-style questions on various aspects of the demonstrated software from participants (N=35).

Question	Strongly disagree, n (%)^a^	Disagree, n (%)^a^	Slightly disagree, n (%)^a^	Neither, n (%)^a^	Slightly like, n (%)^a^	Like, n (%)^a^	Strongly like, n (%)^a^	Unknown, n (%)^b^
Overall appearance	0 (0)	0 (0)	0 (0)	2 (6)	4 (12)	20 (59)	8 (24)	—^c^
Blue beginning scene	0 (0)	0 (0)	0 (0)	1 (3)	2 (6)	20 (57)	11 (31)	1 (3)
Transition through the clouds	0 (0)	0 (0)	0 (0)	2 (6)	2 (6)	20 (59)	9 (26)	1 (3)
Scene around the first door	0 (0)	0 (0)	0 (0)	2 (7)	9 (30)	14 (47)	5 (17)	—
Ground grid	0 (0)	0 (0)	0 (0)	5 (17)	6 (21)	13 (45)	5 (17)	—
Hallway	0 (0)	0 (0)	0 (0)	4 (14)	4 (14)	15 (54)	5 (18)	—
Image size	0 (0)	0 (0)	0 (0)	3 (9)	8 (24)	17 (50)	6 (18)	—
Ball and movement	0 (0)	0 (0)	0 (0)	4 (12)	4 (12)	16 (47)	10 (29)	—
Picture fade	0 (0)	0 (0)	0 (0)	5 (14)	6 (17)	14 (40)	10 (29)	—
Transition to second door and hallway	0 (0)	0 (0)	0 (0)	6 (18)	4 (12)	16 (48)	7 (21)	—
Timing	0 (0)	0 (0)	0 (0)	2 (6)	1 (3)	24 (69)	8 (23)	—
Visual field	0 (0)	0 (0)	0 (0)	2 (6)	2 (6)	18 (53)	12 (35)	—
Flow	0 (0)	0 (0)	0 (0)	2 (6)	2 (6)	18 (51)	13 (37)	—

^a^Results for different response options.

^b^Participants who did not provide a response.

^c^Not applicable.

#### Qualitative Results

Thematic analysis of the open-text survey questions resulted in 3 discovered themes: occupationally tailored virtual environments, individually tailored immersion, and applicability beyond military populations.

##### Occupationally Tailored Virtual Environments

A predominant theme for participants was the need for the 3MDR environment to be occupationally tailored to accurately reflect their specific occupations, needs, and traumatic events. For example, one of the participants noted the following:

...the hallway itself is slightly triggering, hospitals have long hallways with doors that automatically open similarly. So for me I found I was stressed about what I would find behind the doors.

P7

Another participant shared similar concerns for health care providers:

One of my very first impressions is that the maroon color of the flooring in the approach leading up to the hallway immediately reminded me of blood/bodily fluids on the floor such as you might find in response to a patient bleeding or in other trauma situations. This may put people on edge before they even start walking down the hallway toward the actual images, which I am not sure is the desired effect of the study...

P31

Specific professions reported differences in work cultures and commonly encountered traumatic scenarios, expressing the view that 3MDR would need to be adapted to their contexts. For example, a firefighter participant noted the following:

...most Firefighter Stressors are interactions with patients and the death and dying experience rather than Firefighting Operations. Medical Aid events are similar to paramedics and the patient care and the onus of that patient in death and dying.

P36

Similarly, a police participant shared the following:

If there is a way to help members reconcile moral injuries which is prevalent in the policing profession, that would be great.

Another participant noted the following:

...traumatic events in my field often occur when standing still rather than moving or walking, not sure if that would affect the therapy.

P4

Some participants also shared that the “opening scenery, before entering the hallway presents as ominous and foreboding,” which “if deliberate, this is spot on. If not the intent, then something to consider [P19].”

##### Individually Tailored Immersion

The theme of personalization highlighted the need for the 3MDR environment to be both specific and flexible enough that, in addition to being culturally tailored (ie, for individual professions), it could also be adaptable at the personal level. Some of the suggested personal adaptability elements included the possibility of the virtual environment being completely neutral and unrelated to any occupational environment, “Cultural or location centered imagery ie. various hospital settings [ICU, ER, general wards unit] or out of hospital settings [inner city vs. in patient's home, etc]” or “a choice between a nature scene or tunnel” (P2). Equally, it was noted that some participants may struggle with the visual and auditory stimulation of the environment, which would need to be addressed:

I found the hallway movement in the video almost brought me to dissociation [between the movement and the ”lights”]

P35

Another participant noted the following:

...while red stands out and sets a certain tone [floor grid, moving EMDR ball], it is also the most common color to have vision challenges with. The white text in the red balls may be exceptionally challenging for people with some vision impairments. This should be considered as part of the build

P20

This also applied to the use of the harness:

For some people I have worked with, having a harness on could take some getting used to from a sensory perspective. Many members feel constriction in their chest when activated. So there just might be an adaptation period to prepare for

P21

Another participant shared the following:

...any inclusion of various spiritual or faith-based options would be nice

P27

##### Applicability Beyond Military Populations

The final theme identified was large-scale support for 3MDR. Participants reported enthusiasm that 3MDR could have great relevance for populations affected by trauma beyond military members and veterans. For example, one of the participants shared the following:

I am excited to see if this intervention can be researched and disseminated to other communities with trauma, in addition to veterans/safety personnel. For example, folks with a history of sexual trauma or intergenerational trauma from other sources

P16

Participants identified that potentially meaningful populations could include “refugees from war zone, youth from abusive homes, sexual assault victims, youth who had gang involvement” (P12). However, equally, participants noted that frontline PSP and health care providers may be especially vulnerable because of the nature of their work:

Needs to be available to populations beyond the military. Health care and corrections greatly need support like this

P23

For military and first responders, this system is a must have...3MDR could possibly solve issues before they start. I believe with some adjustments to some of the timing issues this could be a viable treatment

P32

Thus, the potential viability of 3MDR in broad populations affected by trauma needs to be explored.

## Discussion

### Principal Findings

Over the past decade, 3MDR has evolved from an intervention requiring physically large and costly infrastructure to more mobile options. The CAREN and GRAIL systems offered an opportunity to trial 3MDR with military members with TR-PTSD using a VRE originally developed for physical rehabilitation. Technologies have since become more mobile, accessible, and cost-effective. Through the research and innovation of this 3MDR international collaboration, the existing hardware and software components of 3MDR were adapted, followed by new components being incorporated, modified, and further validated to enhance the mobility, accessibility, feasibility, and applicability of 3MDR for use with trauma-affected populations. This mixed methods approach, which included a modified Delphi approach, hardware and software development, validation, descriptive statistical analysis, qualitative thematic analysis, and triangulation of the data, allowed the research team to conceptualize, build, and evolve a new 3MDR system. Doing so has positioned 3MDR to be ready for the next wave of populations affected by trauma, which will require novel trauma interventions, including PSP affected by the COVID-19 pandemic.

New hardware and software will benefit from continued studies on feasibility, acceptability, usability, and validity. Although it is expected that the new 3MDR setup will provide similar results to those from previous studies that used the GRAIL, CAREN, and CAREN Lite, new and unpredicted variables could potentially change the experience and outcomes of 3MDR. For example, it remains unknown how patients may react to HoloLens 2 and other wearable metrics and whether this may disrupt the connection between them and their therapist. That said, 3MDR is a protocolized psychotherapeutic intervention. Despite the adaptations and changes in hardware and software, it is not anticipated that deviations from the protocol will result.

### Moving Forward: Hardware

The salient hardware components of the 3MDR compact system were identified through expert consultation. These included the treadmill, safety harness, computer system, treadmill or computer interface, graphics card, visual display, and eye-tracking capability. The characteristics considered included access, physical footprint, portability, therapist positioning, system operation, cost, acceptability, and ease of use. The results yielded a more modular, cost-effective 3MDR compact system and a 3MDR AR system, both of which increased the potential for customized and tailored immersions for populations affected by trauma. The 3MDR compact system comprised a COSMED T150 clinical treadmill, together with a computer built around the Nvidia Quadro P5000 graphics card and short-throw light-emitting diode projectors with display screens [36,37]. Regarding the 3MDR AR system, a blend of virtual and real visual stimuli was determined to be essential, as was the ability of the patient and therapist to effectively see each other and read facial cues. The HoloLens 2 system, together with a laptop, router, and treadmill, were selected [32].

### Moving Forward: Software

Through the modified Delphi consultation process, specific needs were identified that the new 3MDR software would have to meet to reach the goals of enhanced mobility, accessibility, feasibility, and applicability. The team identified (1) ease of adaptation to new technologies, (2) a user-friendly interface, (3) the ability to integrate audio and visual playback components, and (4) data capture and reporting capabilities as priorities for the software. The first was addressed via the integration of Unity 3D to allow for easy adaptation because of its flexibility, modularity, and polymorphism, enabling applications to maintain their relevance with evolving technical innovation. It also offers flexible integration of heart rate monitors, eye trackers, and other wearable devices for future use and can be ported to different devices and displayed using variable means. This would also address the key theme of individualized-tailored immersion, which was recognized by the PSP as a priority in software validation. The UI was designed to be easy for therapists to use, with higher resolution images and rendering. The music selection was integrated into the software. Furthermore, walking speed, notes, and biomarkers can be entered, automatically saved, and easily reviewed. Although many of the aspects incorporated into the software coincided with the results of the software validation study, not all assumptions of the team were matched to the data from the PSP (N=35). Although the research team opted to have a standard VRE display within the software with the music and images being customizable, the PSP expressed a desire to customize the environment to their professions and individual circumstances. The team and the PSP were similar in that they were passionate about 3MDR being adapted and used for other populations affected by trauma. The perceived strengths, weaknesses, and recommendations of the PSP participants will inform further modification and improvement of future 3MDR software.

### Limitations

There were several barriers and limitations that were faced throughout this initiative. The software validation study used a relatively small sample size, and the COVID-19 pandemic necessitated that focus groups be web-based opposed to in person. This meant that some potential participants could not engage because of reduced access to devices; bandwidth; and wired or wireless internet that would allow them to view videos, complete the web-based forms, and engage in focus groups. In addition, pandemic restrictions provided barriers to physically accessing research facilities, which precluded the validation of hardware. In the future, further validation of the hardware and software will be addressed through ongoing consultation with patients, clinicians, and policy makers as further development of 3MDR is pursued.

### Future Directions

Future research and evolution of 3MDR will continue to enhance accessibility and cost-effectiveness while ensuring the effectiveness of 3MDR and patient safety, thereby positioning 3MDR for continued development as technologies emerge. Further study and development will involve populations beyond the military and PSP to include other civilians who have experienced gender-based violence, adverse childhood experiences, natural disasters, global conflicts, intergenerational trauma, and other complex traumas that require novel approaches to rehabilitation and recovery. In addition, 3MDR has traditionally been used in adult populations aged 18 to 65 years. Further studies will involve the efficacy of this intervention for other periods of the life span. Increased mobility and portability will also increase the ability of populations in geographical areas with limited information technology infrastructures, such as remote rural areas and combat zones, to potentially use 3MDR. This may include VR or AR headsets, which are becoming more accessible and common to general consumers. Future studies may also address levels of immersion among different populations affected by trauma with the evolving 3MDR hardware and software to determine whether these modifications influence the efficacy of the intervention.

Future opportunities for 3MDR evolution and research may involve the use of eye tracking and other wearable biosensor systems with the aim of providing therapists with real-time information regarding neurobehavioral indicators and trends. For example, eye-tracking information could provide the therapist with information on the visuoperceptual processing of the patient, including whether they are avoiding certain aspects of the image or neglecting part of the screen and facing challenges tracking the EMDR ball. The use of electroencephalography could potentially measure changes in brain activity to better assess the components of 3MDR that affect neurobehavioral changes. Furthermore, neuroimaging captured before and after the intervention may detect changes resulting from 3MDR.

In addition, increasing the use of narrative approaches, which may include videos, could be implemented to facilitate the impact of the patient’s story. Through tailored immersion, other sensory systems could be integrated into the new 3MDR system to include smells, tactile feedback, environmental sounds, and integration of vestibular and other senses, in addition to visual stimuli. In addition, studies examining the effectiveness of combined treatments to improve the rehabilitation process and timelines should be explored. The consideration of individual and group 3MDR sessions is also worthy of exploration.

### Conclusions

Although military members and veterans continue to exhibit higher rates of PTSD because of their increased exposure to combat and other traumatic scenarios, the effects of PTSD, moral injury, and other occupational stress injuries that continue to disrupt the health, well-being, and quality of life of populations affected by trauma require immediate attention. As an exposure-based, VR-supported therapy, 3MDR is currently being used to treat military members and veterans with TR-PTSD. As this paper has demonstrated, there is both an interest in and a need to adapt the intervention for other populations affected by trauma and improve accessibility to treatment. The aim of this initiative was to enhance the mobility, accessibility, feasibility, and applicability of 3MDR for use with other populations affected by trauma. The resulting hardware and software innovation from this initiative was demonstrated to have some level of construct and face validity and will now be used in further clinical trials of 3MDR with PSP who have been affected by trauma related to the COVID-19 pandemic.

As novel interventions for trauma involving VR and AR may change the traditional therapeutic constellation in the near future, it is of utmost importance to investigate their potential to reduce the symptoms of PTSD and increase the quality of life, daily functioning, and safety of populations affected by trauma, as well as that of their families, wider organizations, and communities. As 3MDR continues to emerge in the literature and shows promise, international efforts are positioning 3MDR for more widespread adoption as a potential first-line treatment for PTSD to add to evidence-based trauma treatments. This has involved the current initiative, which focused on hardware development, software optimization, and validation, as well as facilitation of the adoption of 3MDR in mental health clinics, 3MDR therapist training and certification, and new research directions.

Efforts to evolve and advance 3MDR by further improving mobility, accessibility, feasibility, and applicability will continue to be explored with the goal of continuing to adapt to changing technology, as well as the needs of stakeholders, especially populations affected by trauma.

## Appendix

Multimedia Appendix 1 Focus group instructions and semistructured scripts for the multimodal motion-assisted memory desensitization and reconsolidation software validation study.

## Abbreviations

3MDR: multimodal motion-assisted memory desensitization and reconsolidation
AR: augmented reality
CAREN: Computer Assisted Rehabilitation Environment
EMDR: eye movement desensitization and reprocessing
GRAIL: Gait Realtime Analysis Interactive Lab
HMD: head-mounted display
PSP: public safety personnel
PTSD: posttraumatic stress disorder
REDCap: Research Electronic Data Capture
TR-PTSD: treatment-resistant posttraumatic stress disorder
VR: virtual reality
VRE: virtual reality environment
UI: user interface
