# Supplementary material for: The Redesign and Validation of Multimodal Motion-Assisted Memory Desensitization and Reconsolidation Hardware and Software: Mixed Methods, Modified Delphi–Based Validation Study
Source: JMIR Hum Factors. 2022 Jul 12;9(3):e33682. doi: 10.2196/33682 (PMC9328788; doi:10.2196/33682)
Supplement: Multimedia Appendix 1 [file humanfactors_v9i3e33682_app1.docx]

**Multimedia Appendix 1: Focus Group Instructions and Script**

1. **Introduce yourself and the purpose of the study**

"Hi, my name is _____ and I am a researcher with the Heroes in Mind, Advocacy and Research Consortium, or HiMARC for short, at the University of Alberta. I will be your facilitator today. I am involved in the 3MDR Study which stands for Multi-modal Motion-assisted, Memory Desensitization and Reconsolidation. Currently, this study is taking place at multiple sites around the globe involving military members and veterans with PTSD. In Edmonton, we have received a grant to start trialing this intervention with public safety personnel who have been affected by trauma. Public safety personnel can include first responders, such as police, fire fighters, and emergency medical workers, but also other profession such as dispatchers, nurses, doctors, respiratory therapists, corrections officers, peace officers, border  patrol agents, coast guard personnel, and other professions on the front line of serving the public. We have worked with our international partners to design new software for 3MDR for these professions. The purpose of this meeting today is to gather information on what you, as a public safety personnel, think of the newly designed software. If the results are favourable, this software may be used for our future studies with public safety personnel. You are not obligated to participate in this study. This meeting will not take longer than 30 minutes. Please keep your camera on if possible. Note that you are not permitted to screen capture or record this session"

To start, I would like to show you a couple short videos on 3MDR."

1. **Show the informational 3MDR videos:**

<https://www.youtube.com/watch?v=bD43R_oa6qo>

<https://www.youtube.com/watch?v=bOAbDv-Ai6o&t=3s> - only to 2:35

"Next, if you still would like to participate in this study, please read and fill out the consent form by clicking the link I have posted in the chat. Please let me know if you have any questions. Once you have pressed submit, please DO NOT complete the next form. Just leave your browser with the second survey open, and come back to this chat."

1. **Post the link to the consent form**

Ask the participants to fill it out, press submit, and tell them to leave the browser open

"Now, I would like to show you a video of the newly designed software for public safety personnel. After you have viewed it, please complete the second form in your browser and click submit."

1. **Show the new 3MDR video**

“The link will be emailed to you separately. Please do not share this link under any circumstances. Ask them to fill out the second questionnaire and click submit.

"Thank you for your participation!"
